# Association of *DROSHA* Variants with Susceptibility and Outcomes in Childhood Acute Lymphoblastic Leukemia

**DOI:** 10.3390/cimb47060473

**Published:** 2025-06-19

**Authors:** Ioannis Kyriakidis, Iordanis Pelagiadis, Charalampos Pontikoglou, Helen A. Papadaki, Eftichia Stiakaki

**Affiliations:** 1 Department of Pediatric Hematology-Oncology & Autologous Hematopoietic Stem Cell Transplantation Unit, University Hospital of Heraklion & Laboratory of Blood Diseases and Childhood Cancer Biology, School of Medicine, University of Crete, 71003 Heraklion, Greece; ipelagiadis@icloud.com (I.P.); efstel@uoc.gr (E.S.); 2 Department of Hematology & Hemopoiesis Research Laboratory, School of Medicine, University of Crete and University Hospital of Heraklion, 71500 Heraklion, Greece; xpontik@uoc.gr (C.P.); e.papadaki@uoc.gr (H.A.P.)

**Keywords:** acute lymphoblastic leukemia, childhood, Drosha, microprocessor, microRNA, polymorphism, disease susceptibility, survival, relapse, prognosis

## Abstract

MicroRNAs are key regulators of lymphoid differentiation, exhibiting a pivotal role in acute lymphoblastic leukemia (ALL) biology and prognosis. The initial steps of canonical miRNA biogenesis involve the microprocessor complex processing the primary miRNA transcripts into precursor miRNAs via Drosha. *DROSHA* polymorphisms have been implicated in pediatric ALL and linked with cancer risk. This study investigated the role of rs642321, rs3805500, and rs10035440 *DROSHA* polymorphisms in ALL susceptibility, relapse, and outcomes in children and adolescents of Greek descent. The study included 252 children and adolescents (115 ALL cases and 137 controls). Genotyping was performed using RT-qPCR and the TaqMan Genotyping Assay. Homozygotes for the minor allele in *DROSHA* rs642321 were nominally associated with ALL susceptibility (TT vs. CC+CT; OR 4.5; 95% CI: 1.2–21.2; p_adj_ = 0.034). Likewise, homozygotes for the minor allele in rs3805500 were linked with ALL risk (GG vs. AA+AG; OR 2.7; 95% CI: 1.3–6.1; p_adj_ = 0.012). A suggestive association was observed between the rs3805500 AG genotype and both relapsed (OR 5.8; 95% CI: 1.6–24.3; p_adj_ = 0.011) and deceased cases (OR 5; 95% CI: 1.1–26.3; p_adj_ = 0.038). Patients with the rs3805500 AG and GG genotypes showed a trend toward poorer overall survival rates. In summary, certain haplotypes of *DROSHA* polymorphisms may be modestly associated with the occurrence of childhood ALL and its outcomes, although these findings require validation in larger, independent cohorts.

## 1. Introduction

Acute lymphoblastic leukemia (ALL) is the neoplasia with the highest incidence rates among children and adolescents [1]. Overall survival (OS) in childhood ALL exceeded 90%, mainly due to modern multiagent chemotherapy protocols with balanced intensity based on the stratification according to risk estimators of relapse: age and white blood cell (WBC) count at diagnosis, extramedullary disease, unfavorable risk genetics, and the measurable residual disease (MRD) reflecting the response to therapy [2]. Despite the advancements in risk-adapted protocols, relapse is a significant burden affecting up to 20% of cases, and two-thirds of these patients are expected to succumb to the disease [3]. Beyond the well-characterized genetic defects found in the blasts of specific childhood ALL subtypes, there have been identified inherited genetic variants that play a crucial role in ALL predisposition, drug responsiveness, and therapy-related toxicities [4]. In the same context, various single-nucleotide polymorphisms (SNPs) in microRNA and miRNA-processing genes have been implicated in childhood ALL susceptibility and prognosis [5,6,7].

Over the last three decades, microRNAs (miRNAs or miR) have been in the spotlight due to their role in regulating gene expression, mainly at the post-transcriptional level, by silencing or destabilizing target transcripts. These small non-coding RNA molecules, about 22 nucleotides in length in their mature form, display distinct biological features and target specific genes, and depending on their expression, they act as oncogenes or tumor-suppressive genes [6,8]. Dysregulated miRNA expression contributes to childhood ALL pathogenesis by affecting the cell cycle control balance, apoptosis, and lymphoid differentiation [9,10]. Aberrant miRNA expression profiles have been linked to poor prognosis and drug resistance in ALL. Moreover, miRNAs interact with critical signaling pathways, such as Notch and PI3K/Akt, essential in lymphocyte development and frequently altered in leukemia. Therefore, understanding miRNA involvement in ALL could offer new diagnostic and therapeutic strategies, improving treatment outcomes for affected children [6,11]. The concentration of miRNA levels is regulated in six different levels: (i) production of the primary miRNA transcripts (pri-miRNA); (ii) cleavage of the primary transcript into precursor miRNAs by the Drosha–DGCR8 microprocessor complex (pre-miRNA); (iii) transport of the precursor miRNA from the nucleus to the cytoplasm; (iv) cleavage of the precursor miRNA into its mature functional form by Dicer; (v) interaction with target transcripts, leading to subsequent inhibition or cleavage mediated by the formation of the RNA-induced silencing complex or RISC; and (vi) processes that control the stability and degradation of mature miRNAs [12].

Beyond its role in miRNA biogenesis, the role of the ribonuclease Drosha in childhood ALL has been recently under investigation. Significantly higher levels of *DROSHA* mRNA have been documented in pediatric patients with ALL compared to controls [13]. Moreover, several studies associated *DROSHA* (formerly known as nuclear ribonuclease III or *RNASEN*) variants with childhood ALL susceptibility [14,15,16]. The role of Drosha is essential in the canonical miRNA pathway, and *DROSHA*-knockout has a detrimental effect on miRNA levels (∼16-fold reduction in mature miRNA expression), allowing only the production of Drosha-independent miRNAs and noncanonical miRNAs [17,18]. Drosha independent noncanonical miRNA biogenesis may sustain a low-level miRNA production but requires an intact Dicer RNase [19].

Single-nucleotide polymorphisms (SNPs) affecting miRNA biogenesis or mRNA:miRNA interactions are important risk factors in the development of different types of cancer [20]. The present study aims to investigate *DROSHA* rs642321, rs3805500, and rs10035440 in children and adolescents with ALL and healthy controls from Greece. The selection of the SNPs was based on their location (3′-UTR and intron variants), their functional annotations, and previously published studies. This study is linked to the MICRO-CALLS project (MicroRNAs and Childhood ALL Survival) and was conducted, among other aims, to explore the potential effects of *DROSHA* SNPs on measured miRNA levels.

## 2. Materials and Methods

In this case–control study, 252 subjects were enrolled: 115 children and adolescents with ALL diagnosed and treated in the host institution’s tertiary Pediatric Hematology-Oncology Department, and 137 healthy controls referred for routine checkups. Incident ALL cases were not subjected to exclusion criteria, whereas controls were required to have no family history of cancer. Specimens from the pediatric population with ALL were obtained at remission or at the end of the induction phase in deceased patients. All samples were examined twice. Table 1 describes the characteristics of cases and controls. An a priori power analysis was conducted using G*Power 3.1 Statistical Power Analyses for Mac (Heinrich-Heine-Universität Düsseldorf, Düsseldorf, Germany; https://bit.ly/3ubiGF8, accessed on 16 June 2025). The analysis indicated that a total of 206 participants would be sufficient to detect a statistically significant difference with an odds ratio (OR) of 2.0, α = 0.05, critical z = 1.96, and an actual power of 95.1%.

The legal guardians of each participant signed informed consent forms. Adolescents were also informed at recruitment and asked to sign the respective consent forms. The protocol of this study was approved by the Research Ethics Committee of the University of Crete (number 128/07.11.2023).

Peripheral blood specimens were collected in vials with ethylenediaminetetraacetic acid (EDTA). Genomic DNA was extracted using the PureLink Genomic DNA Mini Kit (Invitrogen, Thermo Fisher Scientific Inc., Waltham, MA, USA). SNPs *DROSHA* rs642321, rs3805500, and rs10035440 were genotyped by TaqMan SNP Genotyping Assays (C___3197426_20, C___3197387_20 and C__11277968_10, respectively; Applied Biosystems, Thermo Fisher Scientific Inc., Life Technologies Corporation, Carlsbad, CA, USA) on Bio-Rad CFX96 Real-Time PCR Detection System (Bio-Rad Laboratories, Hercules, CA, USA), adhering to the manufacturer instructions. A 100% concordance rate was observed among the duplicate sets for each SNP.

The goodness-of-fit chi-squared test assessed the deviation from Hardy–Weinberg equilibrium (HWE) for each SNP in the control group. The two-sided Fisher–Freeman–Halton Exact Test and Pearson Chi-Square test were utilized to evaluate the statistical significance of differences in genotype frequencies between the control group and patients with ALL, as well as the differences in demographic and clinical variables. Logistic regression analysis was performed to calculate ORs and the corresponding 95% confidence intervals (CIs), which were used to estimate the strength of association between the SNPs and ALL risk, adjusted for age and gender. IBM SPSS Statistics version 29.0.1.0 (IBM Corp., Armonk, NY, USA) was employed for the statistical analyses. SNPStats was used to verify the linkage disequilibrium (LD) results, genotype/haplotype distributions, and associations for binary and quantitative responses (https://www.snpstats.net/start.htm, accessed on 16 June 2025; Catalan Institute of Oncology). SNPstats also calculated the Akaike Information (AIC) and the Bayesian Information (BIC) criteria, which determine the goodness-of-fit of an estimated statistical model. The lowest AIC and BIC values identified the recessive model as the best fitting model for all three SNPs [21,22]. LD between *DROSHA* SNP pairs was evaluated utilizing Lewontin’s standardized coefficient D′, and Haploview was used for the presentation of the results [23]. The regulatory motifs altered by each variant, meaning the allele-specific binding affinity changes based on the motifs of key transcriptional regulators, were extracted from VannoPortal (http://www.mulinlab.org/vportal, accessed on 16 June 2025). The influence of each *DROSHA* SNP genotype on gene expression levels was assessed using the Genotype-Tissue Expression (GTEx) Portal (http://www.gtexportal.org, accessed on 16 June 2025). Data meta-analysis was performed using Review Manager version 5.4.1 (RevMan, The Cochrane Collaboration, London, UK). All *p* values are two-sided, and the significance level was set at *p* < 0.05.

## 3. Results

All three polymorphisms in the control samples were in accordance with HWE. Table 2 lists the respective p_HWE_ along with the VannoPortal regulatory effects noted for each polymorphism. The slight male predilection that was calculated for children with ALL (58.3%) was expected [24]. Sex and age did not significantly differ between cases and controls. Table 3 presents the distribution of *DROSHA* rs642321, rs3805500, and rs10035440 genotypes among pediatric patients with ALL and controls and demonstrates the optimal genetic model for each significant association [which, in this case, differs from the additive model typically assessed in genome-wide association studies (GWAS)]. Investigations for all genetic models, the allelic distribution, and crude ORs can be accessed through Table S1.

Homozygosity for the minor allele in *DROSHA* rs642321 was nominally associated with ALL susceptibility (TT vs. CC+CT; OR 4.5; 95% CI: 1.2–21.2; p_adj_ = 0.034). Likewise, homozygosity for the minor allele in rs3805500 was linked with ALL risk (GG vs. AA+AG; OR 2.7; 95% CI: 1.3–6.1; p_adj_ = 0.012). No significant association with childhood ALL risk was calculated for rs10035440 genotypes.

**Figure 1:**
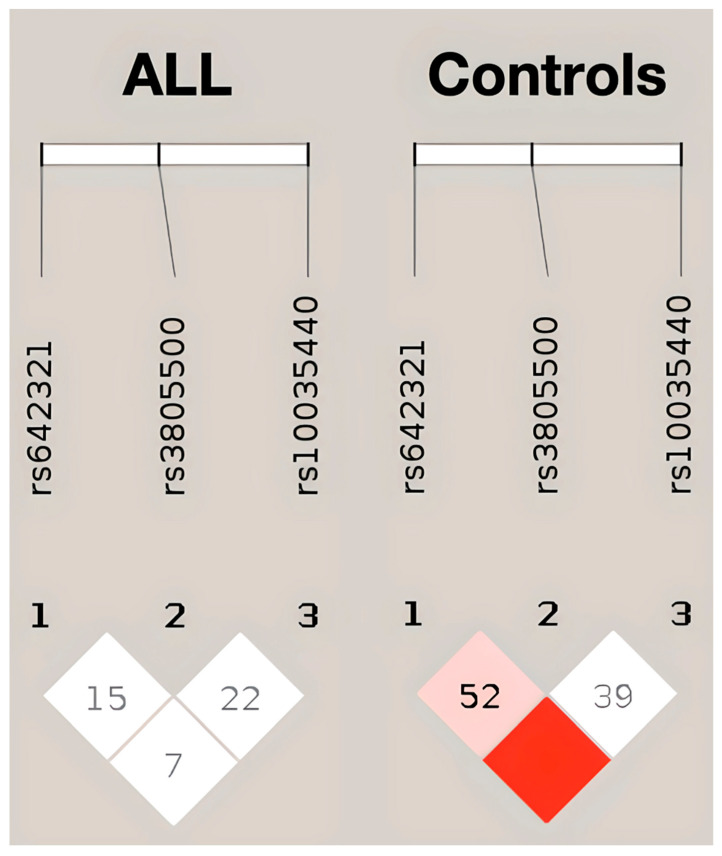
LD plot of *DROSHA* SNPs in patients with ALL (**left**) and controls (**right**), generated using Haploview. Each diamond represents a pairwise LD comparison, with the value inside indicating 100 × D′ (unless D′ = 1.0, in which case the value is omitted). The color gradient reflects the strength of LD: darker red indicates higher D′ values and stronger LD, while white indicates weaker LD.

The linkage disequilibrium analysis of *DROSHA* rs642321, rs3805500, and rs10035440 using Haploview did not indicate strong LD among these variants in patients with ALL, suggesting that rs642321 and rs3805500 may exert independent effects on disease risk (Figure 1). Both D′ and r^2^ values were close to zero, suggesting that none of the SNPs strongly predict each other.

Further analysis of genotypes in-between phenotypes (B- and T-cell) and B-ALL genetic subtypes calculated insignificant correlations. Notably, children with T-ALL had a significantly higher prevalence of homozygosity for the minor TT allele in rs642321 than patients with B-ALL (37.5% vs. 5.9%; *p* = 0.036). The combined TT/GG haplotype (for rs642321 and rs3805500, respectively) did not associate with pediatric ALL diagnosis. The TT/GG haplotype was identified in only one individual from the control group and three patients. The latter finding suggests that the simultaneous heterozygosity for the minor alleles in both SNPs does not likely confer an additional risk for ALL, as supported by the LD analysis above. In addition, haplotype analysis suggested a potentially stronger association with childhood ALL risk for the rs3805500 G allele when chaperoned by the rs642321 C allele, although this finding requires further validation. Table S3 presents the haplotype analysis results for rs642321 and rs3805500. Investigations among patients with ALL indicated significantly higher WBC at diagnosis for GG carriers of rs3805500 (68,540 vs. 31,681; *p* = 0.037). In the same context, homozygotes for the minor allele in rs642321 were diagnosed at an older age (10.95 vs. 6.24; *p* = 0.002). When focusing on ALL subtypes, no patient with hyperdiploidy and all patients with *TCF3*::*PBX1* were homozygotes for the minor allele in rs642321 (Fisher–Freeman–Halton exact test; *p* = 0.051).

**Figure 2:**
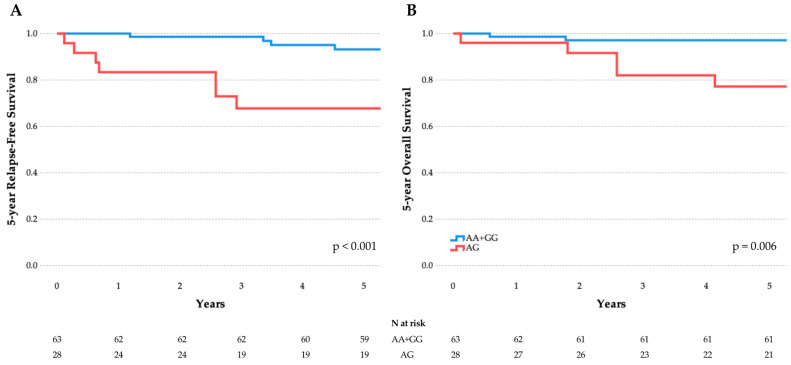
Association of *DROSHA* rs3805500 heterozygotes with RFS (**A**) and OS (**B**) in the childhood ALL cohort. A trend toward lower RFS and OS was observed in AG vs. AA+GG (illustrated here) and AG+GG vs. AA comparisons. These associations remained significant after FDR correction, but did not survive Bonferroni adjustment for multiple testing.

Regarding outcomes, the rs3805500 AG genotype was nominally associated with both relapsed (OR 5.8; 95% CI: 1.6–24.3; p_adj_ = 0.011) and deceased cases (OR 5; 95% CI: 1.1–26.3; p_adj_ = 0.038). Noteworthily, children and adolescents with ALL carrying the rs3805500 AG and GG genotypes showed a trend toward lower OS (84.4%) than those homozygous for the major allele A (98.2%; *p* = 0.014). In the same context, relapse-free survival (RFS) appeared lower in children and adolescents with the rs3805500 AG+GG genotypes (81.8%) than in the AA carriers (94.5%, *p* = 0.048). Figure 2 illustrates the lower 5-year RFS and OS in rs3805500 heterozygotes observed in our cohort. To further evaluate the prognostic impact of rs3805500, a multivariable Cox proportional-hazards model was developed to assess the occurrence of relapse, adjusting for established clinical factors including age and white blood cell count at diagnosis, cytogenetics, and MRD status at the end of induction (day 33). The AG genotype remained an independent predictor of relapse (HR 8.3, 95% CI: 1.2–58.7, *p* = 0.035; detailed description of the model can be found in Table S5), but, as largely anticipated, positive MRD33 was by far the strongest predictor of relapse (HR 15.3; 95% CI: 1.6–143.2; *p* = 0.017). No relapsed or deceased case was found with the CC genotype in rs10035440, but this association failed to reach the significance levels. Haplotype investigations did not reveal significant associations with ALL prognosis.

## 4. Discussion

Regarding *DROSHA* SNPs in whole blood, the investigation of the expression quantitative trait loci (eQTL) with GTEx revealed significant effect sizes. The relevant data can be accessed in Table S2 and Figure S1, both located in the Supplementary Materials. In agreement with previous reports that supported up-regulation of *DROSHA* mRNA in childhood ALL compared to controls, higher predicted Drosha expression was observed among rs642321 TT carriers and rs3805500 GG carriers, genotypes that were suggestively associated with increased susceptibility in this study [13]. Among the transcription factors predicted to exhibit allele-specific binding at the studied DROSHA SNPs, SPI1, TAL1, MYC, and CEBPA stand out because of their established roles in hematopoietic differentiation and leukemogenesis, particularly in ALL [25]. Notably, the T-cell immunophenotype was nominally associated with the risk TT genotype in rs642321, which in turn seems to be associated with a 6-fold increase in affinity change for SPI1 (Table 2).

**Figure 3:**
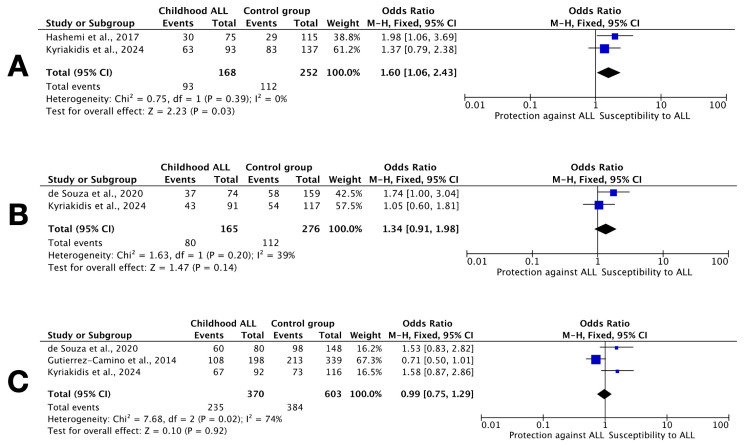
Forest plots of studies including results for *DROSHA* SNPs. (**A**) rs642321 dominant model CC vs. CT+TT; (**B**) rs3805500 dominant model AA vs. AG+GG; and (**C**) rs10035440 dominant model TT vs. CT+CC. Each plot shows the OR and 95% CI for individual studies, including the current Greek cohort [14,15,16]. The summary OR is represented by a diamond. Heterogeneity across studies (I^2^) is indicated. The plots were generated using a random-effects model.

All three SNPs examined in this study have been previously investigated in the context of childhood ALL occurrence, but published results are inconsistent [14,15,16]. A meta-analysis was therefore performed to assess consistency across populations, integrating our findings with those of earlier studies, as illustrated in the forest plots of Figure 3.

Regarding rs642321, a tetra-amplification refractory mutation system (T-ARMS) PCR study from southeast Iran, involving 75 children with ALL and 115 age- and sex-matched healthy children, reported an association with the CC genotype and the dominant model (*p* = 0.037) [14]. In contrast, our study identified a nominal association under the recessive and overdominant models (TT vs. CC+CT, and CC+TT vs. CT, respectively). Despite differing model assumptions, the meta-analysis confirmed a significant overall association with the CC genotype, suggesting that variation in this locus may influence ALL risk (Figure 3A). The low I^2^ value supports cross-study consistency, although dbSNP data indicate that C and T allele frequencies may vary significantly between Iranian and Greek populations (https://www.ncbi.nlm.nih.gov/snp/rs642321, accessed on 16 June 2025).

As regards rs3805500, a study from Brazil genotyped 74 children with B-ALL and 159 adult controls (aged 66 ± 16 years old) and reported a threefold increased risk of ALL for AA carriers (OR 2.9; 95% CI: 1.415–5.998; *p* = 0.004), employing the TaqMan OpenArray Genotyping technology (Real-Time PCR) [15]. However, the latter study’s results warrant interpretation with caution, because they only refer to B-ALL cases, subtype prevalence is far beyond expected (no hyperdiploid cases, 21% with *TCF3*::*PBX1*, 14% with *BCR*::*ABL1*, and only 7% with *ETV6*::*RUNX1*), genotyping failed in 17–19% of cases, and there were significant differences between patients and controls as regards age, sex, and ancestry. In our study, we did not replicate this association (Figure 3B); in fact, we observed a nominal association with the GG genotype instead. Furthermore, when our analysis was restricted to B-ALL cases, no significant association was observed for the AA genotype.

As for the distribution of rs10035440 genotypes, both our study and the previous study from Brazil found no significant genotype distribution differences between patients and controls [15]. However, a study from Spain involving 198 pediatric patients with B-ALL and 339 adult unrelated healthy individuals (aged 51 ± 8 years old), also using TaqMan OpenArray Genotyping technology, reported a significant association of rs10035440 genotypes with susceptibility to B-ALL under the additive genetic model (OR 1.38; 95% CI: 1.04–1.83; *p* = 0.027) [16]. The latter study also examined rs3805500, but did not report any significant findings. Our meta-analysis did not support an association between rs10035440 genotypes and childhood ALL risk (Figure 3C). Taken together, these findings highlight the complexity of genetic contributions to ALL susceptibility and underscore the need for further large-scale, population-specific investigations.

The three studied *DROSHA* SNPs in this study have also been investigated in other types of neoplasia, including chronic lymphoblastic leukemia (CLL). Regarding rs642321, there were no significant associations of any genotype with the risk for breast, lung, or bladder cancer or with lung cancer survival [26,27,28]. As regards rs3805500, there have been indications that the GG genotype could be protective against treatment-related hepatic toxicity experienced by the pediatric ALL population (GG vs. AA+AG; OR 0.12; 95% CI: 0.02–0.98; *p* = 0.009; p_adj_ = 0.239) [29]. Carriers of the GG genotype in rs3805500 may be at greater risk for ALL, but the same population is expected to encounter fewer liver toxicities when diagnosed with the disease. The rs3805500 A allele has also been associated with susceptibility to CLL, while another report associated the rs3805500 minor allele with head and neck cancer prognosis [30,31]. Notably, rs3805500 is in LD with rs640831, a polymorphism previously associated with reduced *DROSHA* mRNA expression and expression changes in 56 miRNAs out of 199 analyzed [28]. Regarding rs10035440, a study in pediatric patients with ALL from Spain reported significantly lower rates of hyperbilirubinemia during induction for the CT+CC carriers (vs. TT; OR 0.23; 95% CI: 0.08–0.68; *p* = 0.0041; p_adj_ = 0.1886) [29]. The rs10035440 has also been correlated with ovarian cancer survival, but results failed to remain significant after false discovery rate (FDR) adjustment [32].

As shown above (Table 2, Table S2, and Figure S1), these three SNPs appear to have putative roles in transcriptional regulation and may influence *DROSHA* expression. Consistent with this hypothesis, higher Drosha levels in rs642321 and rs3805500 homozygotes for the minor allele are anticipated. Significantly higher Drosha expression levels have been previously documented in childhood ALL and CLL specimens compared to healthy controls [13,33]. Deregulated Drosha levels—whether due to regulatory SNPs, structural variants, or deletions—may impair miRNA biogenesis and disrupt the tightly regulated balance between oncogenic and tumor-suppressor miRNAs. This dysregulation may contribute to leukemogenesis by promoting the unchecked proliferation, impaired apoptosis, and altered differentiation of lymphoid progenitor cells. Similar mechanisms have been implicated in other malignancies, including lung, breast, skin, bladder, and head and neck cancers [16,31,34]. Moreover, the examined intronic and 3′-UTR variants may also influence alternative splicing, potentially affecting the subcellular localization of Drosha and impacting non-canonical miRNA processing pathways [35].

Possible limitations of the present case–control study are as follows: (i) its sample size, although a priori analysis showed that the study population is sufficient to deduce significant correlations with actual power exceeding 95%; (ii) the descent of the population from Crete, a confined island region of Greece, although no deviations from the HWE were observed in the control group; (iii) analytical type limitations, referring to whether observed significant differences can explain differences between patients and controls. The study lacks the rigor to determine whether findings are causal or affected by confounding variables, although the respective statistical analyses detected no such issues. After applying the Bonferroni correction for multiple testing (α = 0.0056), none of the associations between the three *DROSHA* SNPs and ALL remained statistically significant. Furthermore, no relevant GWAS study indicated a potential role for *DROSHA* variants (https://www.ebi.ac.uk/gwas/efotraits/MONDO_0000870, accessed on 16 June 2025). However, when applying a less stringent FDR correction with Benjamini–Hochberg procedure, five associations—including rs642321 (recessive and overdominant models) and rs3805500 (recessive, homozygote, and relapse risk)—remained statistically significant (*q* < 0.05; details in Table S4), supporting the potential relevance of these findings. Moreover, the associations observed for rs3805500 and rs642321 in reports from similar case–control studies may justify further investigation in larger cohorts.

Future research could benefit from expression analysis of Drosha in these specimens and further investigations using RNA-sequencing. Quantifying microRNAs and other microRNA-processing partners may also provide deeper insights into the involved molecular mechanisms [36]. In the context of studying the canonical miRNA biogenesis pathway, recent data support the association of minor G allele carriers in *DICER1* rs3742330 and minor allele A homozygotes of *AGO1* rs636832 with childhood ALL risk [37].

## 5. Conclusions

Nominal associations were observed between *DROSHA* rs642321 and rs3805500 and ALL susceptibility in children and adolescents from Crete, Greece. The present case–control study is the first to investigate these SNPs in a population of Greek descent and only the fourth to examine them in childhood ALL. A trend toward increased ALL risk was observed in rs642321 TT carriers, and rs3805500 GG carriers also showed a potential increase in susceptibility. A meta-analysis incorporating previous studies supported a significant overall association for rs642321, suggesting a possible role in ALL predisposition. Statistical analysis disclosed the increased prevalence of high-risk ALL features among the homozygotes for the minor allele in rs642321 (higher age at diagnosis and T-cell immunophenotype) and rs3805500 (higher WBC at diagnosis). Furthermore, the alternative allele in rs3805500 was more prevalent among relapsed and deceased cases. These preliminary findings may reflect a modest contribution of miRNA-processing gene polymorphisms to ALL biology. Further validation in different ethnic groups, larger and independent cohorts, is essential to establish the clinical relevance of these associations.

## Figures and Tables

**Table 1:** Participant characteristics.

Trait	Patients with ALL (n = 115)	Control Group (n = 137)
Median age (Range)	4.9 (1.2–18.1)	4.2 (0.5–18.5)
Sex		
Female	48 (41.7%)	69 (50.4%)
Male	67 (58.3%)	68 (49.6%)
Relapse	15 (13%)	
Overall survival	106 (92.2%)	
Median WBC at diagnosis	13,000/μL	
(Range)	(800–498,000)	
Lineage		
B	103 (89.6%)	
T	12 (10.4%)	
Cytogenetics		
Hyperdiploidy	36 (31.3%)	
*ETV6*::*RUNX1*	34 (29.5%)	
Monosomies and near-diploid ALL	21 (18.3%)	
*BCR*::*ABL1*	4 (3.5%)	
*TCF3*::*PBX1*	3 (2.6%)	
*ΚΜΤ2A*-r	1 (0.9%)	
Other	16 (13.9%)	
High-risk stratification	43 (37.4%)	
Positive MRD on day 33	31 (27%)	

**Table 2:** HWE and regulatory effects of the SNPs in the *DROSHA* gene.

Variant	Position GRCh38.p14	Global Frequency	European Frequency	p_HWE_	Allele-Specific Binding Affinity Changes	Location
rs642321	chr5:31400896	T = 21.1% C = 78.9%	T = 19.5% C = 80.5%	0.929	SOX6, CEBPA, THAP1, CDK2, MAFK, MEF2B, CUX1, RUNX2, SRF, FOXA1, SMARCA1, SPI1, FOXA3, HOXA1	3′-UTR variant
rs3805500	chr5:31462870	G = 41% A = 59%	G = 32.9% A = 67.1%	0.469	TAL1, SPI1, NR4A1, IRF2, HEY1, MYC, SOX2, ESRRA, FOXA2, CTCF, IRF5, NR2C1, AR, POU2F2, ZNF274, BRF1	Intron variant
rs10035440	chr5:31539356	T = 82% C = 18%	T = 81.5% C = 18.5%	0.682	CEBPA, HSF2, NANOG, BCL2, IRF2, CUX1	C5orf22 intron variant

The frequencies correspond to data obtained from the ALFA project in dbSNP (https://www.ncbi.nlm.nih.gov/snp/, accessed on 16 June 2025). ChIP-seq analysis reveals overlapping binding peaks of NKX2-1 and GATA3 at rs3805500, as well as of FOXA1, FOXA2, AR, and HOXB13 at rs10035440 across various tissues (http://www.mulinlab.org/vportal, accessed on 16 June 2025).

**Table 3:** Summary of nominal genotype associations between *DROSHA* SNPs rs642321, rs3805500, and rs10035440 and susceptibility to ALL in children from Crete, Greece.

**SNP**	**Genotype**	**Controls**	**Cases**	***p***	**Best Model**	**OR (95% CI)**	***p***
rs642321	CC	83 (60.6%)	63 (67.7%)	0.037	Recessive TT vs. CC+CT	4.467 (1.202–21.216)	0.034
	CT	51 (37.2%)	23 (24.7%)				
	TT	3 (2.2%)	7 (7.5%)				
rs3805500	AA	54 (46.2%)	43 (47.3%)	0.035	Recessive GG vs. AA+AG	2.734 (1.269–6.118)	0.012
	AG	51 (43.6%)	28 (30.8%)				
	GG	12 (10.3%)	20 (22%)				
rs10035440	TT	73 (62.9%)	67 (72.8%)	NS	NS results		
	CT	36 (31%)	22 (23.9%)				
	CC	7 (6%)	3 (3.3%)				

NS = not significant.

## Data Availability

The data presented in this study are available upon request from the corresponding author for privacy reasons.

## Supplementary Materials

The supporting information can be downloaded at: https://www.mdpi.com/article/10.3390/cimb47060473/s1.

## Abbreviations

AIC	Akaike information criteria
BIC	Bayesian information criteria
ALL	Acute lymphoblastic leukemia
ChIP-seq	Chromatin immunoprecipitation sequencing
CI	Confidence interval
EDTA	Ethylenediaminetetraacetic acid
FDR	False discovery rate
GTEx	Genotype-Tissue Expression
GWAS	Genome-wide association study
HWE	Hardy–Weinberg equilibrium
LD	Linkage disequilibrium
MRD	Measurable residual disease
NS	Not significant
OR	Odds ratio
OS	Overall survival
PCR	Polymerase chain reaction
RFS	Relapse-free survival
SNP	Single-nucleotide polymorphism
UTR	Untranslated region
WBC	White blood cell
