# Supplementary material for: Association of DROSHA Variants with Susceptibility and Outcomes in Childhood Acute Lymphoblastic Leukemia
Source: Curr Issues Mol Biol. 2025 Jun 19;47(6):473. doi: 10.3390/cimb47060473 (PMC12191901; doi:10.3390/cimb47060473)
Supplement: Supplementary file 1 [file cimb-47-00473-s001.zip › cimb-3691805-supplementary.pdf]

Supplementary Materials

Association of *DROSHA* Variants with Susceptibility and Outcomes in Childhood Acute Lymphoblastic Leukemia

Table S1. Associations between *DROSHA* SNPs rs642321, rs3805500, and rs10035440 and susceptibility to ALL in children from Crete, Greece.

DROSHA rs642321							
Genotype	Cases (n = 93)	Controls (n = 137)	p	Crude OR (95 % CI)	p	Adjusted OR (95 % CI)	p
CC	63 (67.7 %)	83 (60.6 %)	0.037	1.00		1.00	
CT	23 (24.7 %)	51 (37.2 %)		0.594 (0.325-1.065)	0.084	1.171 (0.542-2.498)	0.684
TT	7 (7.5 %)	3 (2.2 %)		3.074 (0.820-14.697)	0.114	4.271 (0.634-40.992)	0.147
Allelic model							
C	149 (80.1 %)	217 (79.2 %)	0.812				
T	37 (19.9 %)	57 (20.8 %)					
Genetic model							

Dominant (CC vs. CT+TT)				1.366 (0.789-2.392)	0.269	1.073 (0.935-1.236)	0.320
Recessive (TT vs. CC+CT)				3.636 (0.982-17.219)	0.067	4.467 (1.202-21.216)	0.034
Over-dominant (CC+TT vs. CT)				1.805 (1.014-3.279)	0.048	1.082 (1.008-1.166)	0.033
Homozygote (TT vs. CC)				3.074 (0.820-14.697)	0.114	4.189 (0.595-41.689)	0.162
Heterozygote (CT vs. CC)				0.594 (0.325-1.065)	0.084	0.962 (0.832-1.109)	0.590
Additive (per copy of allele not-C vs. C)				1.042 (0.602-1.804)	0.882	0.985 (0.560-1.734)	0.958
DROSHA rs3805500							
Genotype	Cases (n = 91)	Controls (n = 117)	p	Crude OR (95 % CI)	p	Adjusted OR (95 % CI)	p
AA	43 (47.3 %)	54 (46.2 %)	0.035	1.00		1.00	
AG	28 (30.8 %)	51 (43.6 %)		0.689 (0.372-1.266)	0.233	0.628 (0.262-1.458)	0.286
GG	20 (22 %)	12 (10.3 %)		2.093 (0.933-4.863)	0.078	2.947 (1.045-8.761)	0.044
Allelic model							

A	114 (62.6 %)	159 (67.9 %)	0.258				
G	68 (37.4 %)	75 (32.1 %)					
Genetic model							
Dominant (AA vs. AG+GG)				1.045 (0.603-1.811)	0.514	0.958 (0.460-1.992)	0.908
Recessive (GG vs. AA+AG)				2.465 (1.148-5.495)	0.023	2.734 (1.269-6.118)	0.012
Over-dominant (AA+GG vs. AG)				1.739 (0.982-3.117)	0.060	1.790 (0.840-3.925)	0.137
Homozygote (GG vs. AA)				2.093 (0.933-4.863)	0.078	2.874 (1.025-8.519)	0.049
Heterozygote (AG vs. AA)				0.636 (0.265-1.475)	0.298	0.689 (0.372-1.226)	0.233
Additive (per copy of allele not-A vs. A)				1.099 (0.697-1.735)	0.684	1.093 (0.684-1.745)	0.711
DROSHA rs10035440							
Genotype	Cases (n = 92)	Controls (n = 116)	p	Crude OR (95 % CI)	p	Adjusted OR (95 % CI)	p
TT	67 (72.8 %)	73 (62.9 %)	0.315	1.00		1.00	

CT	22 (23.9 %)	36 (31 %)		0.666 (0.353-1.237)	0.203	0.949 (0.416-2.125)	0.900
CC	3 (3.3 %)	7 (6 %)		0.467 (0.098-1.754)	0.284	0.663 (0.054-5.056)	0.705
Allelic model							
T	156 (84.8 %)	182 (78.4 %)	0.100				
C	28 (15.2 %)	50 (21.6 %)					
Genetic model							
Dominant (TT vs. CT+CC)				1.579 (0.876-2.885)	0.132	1.160 (0.540-2.535)	0.705
Recessive (CC vs. TT+CT)				0.525 (0.111-1.947)	0.360	0.659 (0.054-4.966)	0.700
Over-dominant (TT+CC vs. CT)				1.432 (0.775-2.689)	0.256	1.000 (0.450-2.260)	1.000
Homozygote (CC vs. TT)				0.467 (0.098-1.754)	0.284	0.679 (0.059-5.062)	0.715
Heterozygote (CT vs. TT)				0.666 (0.353-1.237)	0.203	0.952 (0.418-2.132)	0.905
Additive (per copy of allele not-T vs. T)				1.306 (0.766-2.229)	0.327	1.360 (0.794-2.327)	0.263

Table S2. The cis-eQTL values of the three *DROSHA* SNPs in whole blood according to GTEx.

SNP	P-Value	Normalized Effect Size	T-statistic
rs642321	0.00038	-0.078	-3.6
rs3805500	0.026	-0.043	-2.2
rs10035440	0.028	-0.051	-2.2

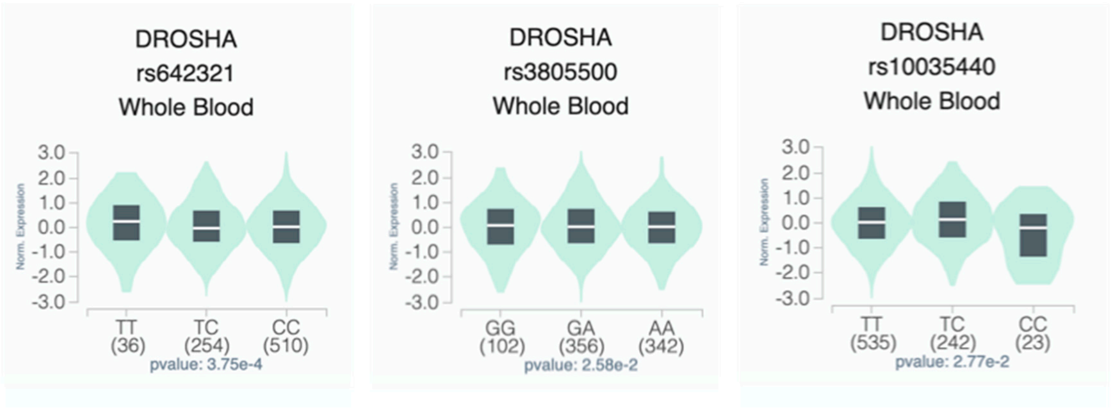

Figure S1. The eQTL violin plots for each SNP genotype according to GTEx.

Table S3. Haplotype analysis for rs642321 and rs3805500.

rs642321	rs3805500	Controls	ALL	Total	Cum. Freq.	OR	p
C	A	0.6149	0.5214	0.5758	0.5758	1.00	-
C	G	0.1771	0.2752	0.2144	0.7901	1.73 (1.03 – 2.89)	0.039
T	G	0.1409	0.097	0.1265	0.9166	0.79 (0.39 – 1.62)	0.52
T	A	0.0671	0.1064	0.0834	1	1.81 (0.82 – 4.02)	0.14

Table S4. False discovery rate and Bonferroni correction results for *DROSHA* SNP associations with ALL susceptibility and outcomes.

Test	p-value	FDR q-value	Sig. at FDR 0.05	Sig. at Bonferroni α = 0.0056
rs642321 Recessive model	0.034	0.049	Yes	NS
rs642321 Overdominant model	0.033	0.049	Yes	NS
rs3805500 Recessive model	0.012	0.042	Yes	NS
rs3805500 Homozygote model	0.049	0.049	Yes	NS
rs3805500 AG vs. relapse	0.011	0.042	Yes	NS
OS for rs3805500 AG+GG vs. AA	0.014	0.050	NS	NS
RFS for rs3805500 AG+GG vs. AA	0.048	0.050	NS	NS
Haplotype rs642321C / rs3805500G	0.039	0.050	NS	NS

Table S5. Multivariable Cox proportional-hazards model for relapse-free survival in patients with childhood ALL, including rs3805500 genotype and established prognostic factors.

	B	SE	Wald	Sig.	Exp(B)	95.0% CI for Exp(B)	
						Lower	Upper
rs3805500 AG	2.111	1.001	4.450	0.035	8.255	1.162	58.666
Age <1 or >10 years	1.929	1.036	3.463	0.063	6.880	0.903	52.451
WBC > 50,000/μL	0.121	0.985	0.015	0.902	1.128	0.164	7.772
Positive MRD33	2.729	1.140	5.731	0.017	15.323	1.640	143.155
Cytogenetic group	-0.120	0.219	0.302	0.583	0.887	0.578	1.361
